# The Overexpression of eIF4E Decreases Oxytocin Levels and Induces Social Cognitive Behavioral Disorders in Mice

**DOI:** 10.1523/ENEURO.0387-24.2024

**Published:** 2024-12-03

**Authors:** Juan Wang, Sijie Chen, Miao Zhao, Lizhen Zheng, Xinxin Huang, Xin Hong, Jie Kang, Ping Ou, Longsheng Huang

**Affiliations:** ^1^College of Clinical Medicine for Obstetrics & Gynecology and Pediatrics, Fujian Medical University, Fuzhou 350001, China; ^2^Child Health Center, Fujian Maternity and Child Health Hospital, Fuzhou 350001, China; ^3^Rehabilitation Department, Fujian Maternity and Child Health Hospital, Fuzhou 350001, China; ^4^Department of TCM Syndrome Research Base, Fujian University of Traditional Chinese Medicine, Fuzhou 350122, China

**Keywords:** cognitive behavioral disorder, eIF4E, hippocampus, oxytocin

## Abstract

Overexpression of the eukaryotic initiation factor 4E (*eIF4E*) gene has been associated with excessive stereotypic behaviors and reduced sociability, which manifest as autism-like social cognitive deficits. However, the precise mechanisms by which *eIF4E* overexpression insufficiently induces these autism-like behaviors and the specific brain regions implicated remain insufficiently understood. Oxytocin (OXT), a neurotransmitter known for its role in social behavior, has been proposed to modulate certain autism-related symptoms by influencing microglial function and attenuating neuroinflammation. Nonetheless, the contributions of the hippocampus and oxytocin in the content of *eIF4E* overexpression-induced autistic behaviors remain elucidated. To investigate this issue, researchers utilized the three-chamber social interaction test, the open-field test, and the Morris water maze to evaluate the social cognitive behaviors of the two groups of mice. Additionally, ELISA, immunofluorescence, Western blotting, and qRT-PCR were employed to quantify oxytocin levels and assess hippocampal microglial activation. The results indicate that overexpression of *eIF4E* in mice is associated with significant impairments in social cognition, alongside pronounced marked hyperactivation of hippocampal microglia.

## Significance Statement

Autism spectrum disorder encompasses a range of neurodevelopmental disorders characterized by social cognitive impairment. Research has indicated a correlation between the overexpression of *eIF4E* gene and autism-like social cognitive impairment. Oxytocin, a neurotransmitter, plays a role in regulating hippocampal microglial activity and attenuating neuroinflammation. This modulation may impact social cognition in individuals with autism. Nevertheless, it remains unclear whether there is an involvement of the hippocampus and OXT in autism-like social cognitive impairments due to *eIF4E* overexpression. The present study suggests that overexpression of *eIF4E* may induce hyperactivation of microglia and contribute to social cognitive impairment by decreasing oxytocin levels in the hippocampus. These findings offer molecular insights into the manifestation of autism-like behavior resulting from *eIF4E* overexpression and may guide future clinical interventions.

## Introduction

Autism spectrum disorder (ASD) is a complex neurodevelopmental disorder characterized primarily by social cognitive deficits and repetitive stereotypical behaviors (Zeidan et al., 2022). According to the US Centers for Disease Control and Prevention, the prevalence of ASD was 2.8% in 2023, with a global prevalence of 1 in 36 (Kodak and Bergmann, 2020; Maenner et al., 2023). This condition has a profound impact on the physical and mental health of those affected children and significantly affects their quality of life. Despite extensive research to elucidate the pathogenesis of social cognitive impairment in ASD, the underlying mechanisms remain incompletely understood.

The hippocampus, a key center for memory storage, plays a critical role in social cognitive functions. The structural and functional integrity of the hippocampus is essential for normal social cognition, and damage to this region is closely linked to the development of social cognitive impairments (Basu et al., 2024). There is increasing evidence that genetic factors are major factors in the manifestation of social cognitive deficits in individuals with ASD. Among these genetic factors, the eukaryotic initiation factor 4E (*eIF4E*) has garnered significant attention (Gkogkas et al., 2013). Epigenomic analysis of white blood cell DNA in newborns with autism has revealed elevated expression of the eIF4E gene compared with those without autism (Bahado-Singh et al., 2019).

In addition, previous animal studies (Sharma and Mehan, 2021) have shown that transgenic mice overexpressing *eIF4E* exhibit abnormal hippocampal neuronal functions and behaviors resembling autism-like social cognitive impairments. Xu et al. (2020) also found that overexpression of eIF4E leads to microglial dysfunction by inducing a transition from a resting state to an activated state, leading to impaired sociability, a hallmark of autism. However, the precise mechanisms by which *eIF4E* overexpression affects microglial function remain to be fully elucidated.

Oxytocin (OXT), a neuropeptide and hormone that is evolutionarily conserved, is known for modulating social behaviors and the female reproductive system. Previous studies have implicated that mutations in the oxytocin gene may contribute to social cognitive deficits. For instance, mutations in Cntnap2 (J. Zhang et al., 2023), a gene associated with ASD in humans, have been shown to reduce oxytocin levels in mouse models. In contrast, exogenous oxytocin administration has been shown to influence social behavior in these models (Gkogkas et al., 2013). Numerous studies have documented the potential of oxytocin to ameliorate social cognitive impairments in individuals with ASD (Higashida et al., 2024; R. Liu et al., 2024). Intranasal administration of oxytocin has been shown to reduce microglial activation (Meng et al., 2023), though further research is needed to ascertain whether this reduction directly correlates with improvements in social cognition.

Our study provides evidence of decreased oxytocin expression in the hippocampus and overactivation of microglia in *eIF4E*-overexpressing mice. This microglial activation may be associated with the reduced oxytocin levels observed. Our results suggest that reduced oxytocin levels may be a factor contributing to the development of autism-like behaviors due to overexpression of *eIF4E*.

## Materials and Methods

### Animals and measured parameters

eIF4E^ki^^/ki^ mice of C57Bl/6J background were purchased from Jiangsu Saye Biotechnology. The mice were backcrossed with C57Bl/6J mice for >10 generations. Heterozygous crosses of littermates were used in all experiments. The use of control mice corresponds to gene typing in mice with mice. Pups remained with their mothers until they were weaned on postnatal day 21, after which they were divided into same-sex groups (up to four per cage) or pair groups (for family socialization).

The experimental animals were housed in a controlled environment at the experimental animal center of the local university, where adequate food and water were provided. The temperature was maintained at (21 ± 1)°C and humidity at 60%, and the light hours were from 7:00 A.M. to 7:00 P.M. In order to investigate the adolescent incidence of ASD and the higher prevalence of ASD in males (H. Li et al., 2024; Leow et al., 2024), we selected two groups of 4-week-old male mice for experiments. Mice that exhibited* eIF4E* heterozygous expression or premature death were excluded from the study, but the remaining mice were included. The exclusion criteria comprised heterozygote *eIF4E* expression and premature death. All the other mice were deemed to meet the requirements of the included studies. This study did not identify any confounding factors. The allocation of mice during the experimental phase is known only to the experimenter, who is responsible for ensuring that the randomization process is properly carried out. The experiment was conducted on two groups of mice, each comprising 12 individuals with distinct eIF4E^ki/ki^ (eIF4E^ki/ki^ group) and wild genotypes (control group). Following genetic identification at Week 4, the mice were subjected to behavioral testing and subsequently euthanized for molecular analysis.

For tissue preparation, mice were weighed and anesthetized with 3% sodium pentobarbital at a dose of 45 mg/kg. After collecting blood samples from the apex with a capillary needle, mice were quickly infused with approximately 120 ml of prechilled 0.9% sodium chloride injection using a syringe until the liver paled. Six brain tissues were then selected for biochemical analysis and immediately frozen in liquid nitrogen. In contrast, the remaining three groups of brain tissue were fixed in 4°C paraformaldehyde for subsequent immunohistochemical experiments.

The detailed molecular assays are described below. All experimental procedures were approved by the local ethical commission and conducted in full compliance with the guidelines of the EU directive on the protection of animals used for scientific purposes (2010/63/EU).

### Genotyping

According to the eIF4E overexpression sequence provided by Sail Biological Company, genotyping was performed using the following primers: standard mouse primer (519 bp), forward 5′-CTCTACTGGAGGAGGACAAACTG-3′, reverse 5′-GTCTTCCACCTTTCTTCAGTTAGC-3′; primers for eIF4E mutation (298 bp): forward 5′-GATGCAGTCACACACATAGGGAGGG-3, reverse 5′-CTTTATTAGCCAGAAGTCAGATGC-3′. Tails of mice (0.4–0.6 cm) from 4-week-old pups bred from the eIF4E overexpression mice were collected and placed in a sterilized centrifuge tube (1.5 ml).

In accordance with the instructions provided with the kit, genomic DNA was extracted using a rapid mouse tail identification kit. The target band was amplified through a PCR and visualized by electrophoresis under long-wave UV light. The final development results were observed to ascertain whether the mice were homozygous, a prerequisite for subsequent experiments.

### Three-box social experiment

The three-chamber social preference test (TCSP) is a method of assessing social behavior, cognition, and memory in animals (Erdogan et al., 2023; Aboubakr et al., 2023). The adaptation stage involved placing the mice in the central chamber of the behavioral apparatus with both small doors open for 10 min to acclimate them to the environment. The social ability test was then conducted with the mice returned to the central chamber while both side doors were closed. A randomly selected individual of the same species, sex, and age was introduced into one compartment, while the other was left vacant. Subsequently, the door was opened, and the mouse's social behavior during a 10 min free activity period was recorded. The social novelty test is as follows: After completing the social ability test, the mice were once again placed in the central chamber with all doors closed. The Stranger Mouse 1 was confined to its respective compartment while another Stranger Mouse 2 of identical species, sex, and age was introduced into an empty restraint.

### Self-grooming experiment

Self-grooming is an observational method for studying spontaneous repetitive activities in mice (X. Fu et al., 2024). For this experiment, mice were placed in a clean standard test box with an observer 2 m away from the box. Before starting, the mice were given 10 min to adapt to the test environment. During this time, the number and duration of each mouse's self-grooming behavior was recorded.

### Open-field experiment

The open-field test (OFT) is a widely used experimental methodology in the fields of neuropsychiatric disorders and neuropsychopharmacology. The test is used to assess learning and memory abilities in mice and to detect their social behavior and anxiety levels (Teneqexhi et al., 2023). The apparatus employed for the test was a white acrylic box measuring 45 cm × 45 cm × 30 cm. The mice were placed in the center of the apparatus and allowed to explore freely for a period of 10 min. Their position was monitored continuously using tracking software, namely Top Scan Lite. The number of times the mice entered the central area of the open field and their duration of stay was automatically recorded and measured throughout the task. The open-field center was defined as an interior area measuring 20 cm × 20 cm.

### Morris water maze test

The Morris water maze is a well-established behavioral assay for assessing spatial learning and memory in animals (Hooshmandi et al., 2021). It is a frequently utilized method for investigating cognitive processes related to learning and memory. During the experiment, mice in each group underwent the Morris water maze localization navigation test over a period of 5 d, from Day 1 to Day 5. The water maze was segmented into four quadrants, each with designated entry points. The time taken by the experimental animals to reach the hidden platform from each entry point was recorded, with a maximum allowed duration of 60 s per trial, referred to as escape latency. Mice that were unable to locate the platform were guided toward it and allowed to remain there for 15 s to cultivate their spatial positioning navigation memory ability before undergoing training at an alternative entry point. On the sixth day of the experiment, the platform was removed, and the mice were tested at any selected entry point. The mice were required to enter the target quadrant, cross over onto the platform, remain in the target quadrant for a specified time (within 60 s), and demonstrate a swimming speed within the acceptable range.

### Immunofluorescence

The hippocampal sections of mice were air-dried and fixed with paraformaldehyde. A mixture of secondary antibodies (1:100, Abcam, ab150080), labeled with 594 sheep anti-rabbit IgG (eIF4E: 1:300, Abcam, ab33768) was applied and incubated for 45 min at room temperature. The slides were counterstained with an anti-fluorescence quenching solution containing DAPI. Subsequently, the images were acquired using a fluorescence microscope, and the average fluorescence intensity was calculated with Image-Pro Plus 6.0 software. Fluorescence double staining was employed to determine microglial activation and eIF4E expression within microglia. Additionally, colocalization analysis was performed to assess the degree of overlap between microglia and eIF4E.

### ELISA

The hippocampal tissue was homogenized using a high-speed homogenizer, and the resulting supernatant was collected following centrifugation. An ELISA kit was employed to measure the expression levels in both the hippocampus and serum samples. The detection procedure was conducted using a DR-200Bs microplate reader, in strict accordance with the instructions provided.

### Quantitative real-time PCR

The hippocampal total RNA was isolated using the LS1040 ki, and its levels and integrity were assessed using a UV-9000 UV spectrophotometer. DNase-treated total RNA was subjected to qRT-PCR analysis on a LightCycler 2.0 instrument using Hieff qPCR SYBR Green Master Mix. The expression levels of OXT, eIF4E, and Iba-1 mRNA were determined by the 2^−ΔΔ Ct^ method normalized to β-actin as an internal control reference gene (primer sequences: OXT forward 5′-GATATGCGCAAGTGTCTCCC-3′, reverse 5′-GCGCGCTAAAGGTATTCCCA-3′; eIF4E forward 5′-ACTGTCGAGTCGCGTCCA-3′, reverse 5′-ATCCATGGCGAACTGGTGG-3′; Iba-1 forward 5′-AGGAGATTTCAAAAGCTGATGTGG-3′, reverse 5′-GACGCTGGTTGTCTTAGGCTGA-3′; CD68 forward 5′-CGTTACTCTCCTGCCATCCTC-3′, reverse 5′-ACATTTCCGTGACTGGTGGT-3′, GAPDH forward 5′-ACGGCAAGTTCAACGGCACAG-3′, reverse 5′-GAAGACGCCAGTAGACTCCACGAC-3′).

### Western blot

The total protein concentration in brain tissue samples was analyzed using the BCA protein assay kit. Subsequent electrophoresis was performed at 80 ν for 1 h using the Omni-Easy PAGE Gel Rapid Preparation kit. Following electrophoresis, the proteins were transferred to the membrane using the Trans-Blot Turbo. The total proteins were probed with antibodies against eIF4E (1:500, Abcam, ab33766), Iba-1 (1:500, Abcam, ab178864), OXT (1:500, Abcam, ab20973), cluster of differentiation 68 (CD68, 1:1000, Abcam, ab283654), and β-tubulin (1:1,000, Cell Signaling Technology, 2146S), respectively. After incubation overnight at 4°C with primary antibodies, goat anti-mouse monoclonal antibody (Abcam, ab130798, 1:1,000) and goat anti-mouse monoclonal antibody (Abcam, ab136716, 1:1,000) were applied at room temperature for 2 h followed by exposure to ECL chemiluminescence solution. For statistical analysis, ImageJ software was used to analyze the gray values of eIF4E, OXT, CD68, and Iba-1 proteins relative to the internal reference β-tubulin. Analysis was performed using Image Lab software.

### Statistical analysis

Behavioral analysis software was utilized to analyze the behavior of mice. Data analysis was performed using SPSS 26.0 statistical software. Measurement data were presented as mean ± standard deviation $\left(\bar{\chi }\pm S\right)$. The normal distribution of the data was tested using the D’Agostino and Pearson’s test. Data were analyzed by one-way ANOVA with Fisher's LSD test or Kruskal–Wallis test with uncorrected Dunn's test according to whether the distribution was normal or not, respectively. A value of *p* < 0.05 was considered as significant. All statistical calculations and graphs were performed using GraphPad Prism software (version 8.4.2).

## Results

### Overexpression of eIF4E leads to social cognitive impairment in mice

**Figure 1.**
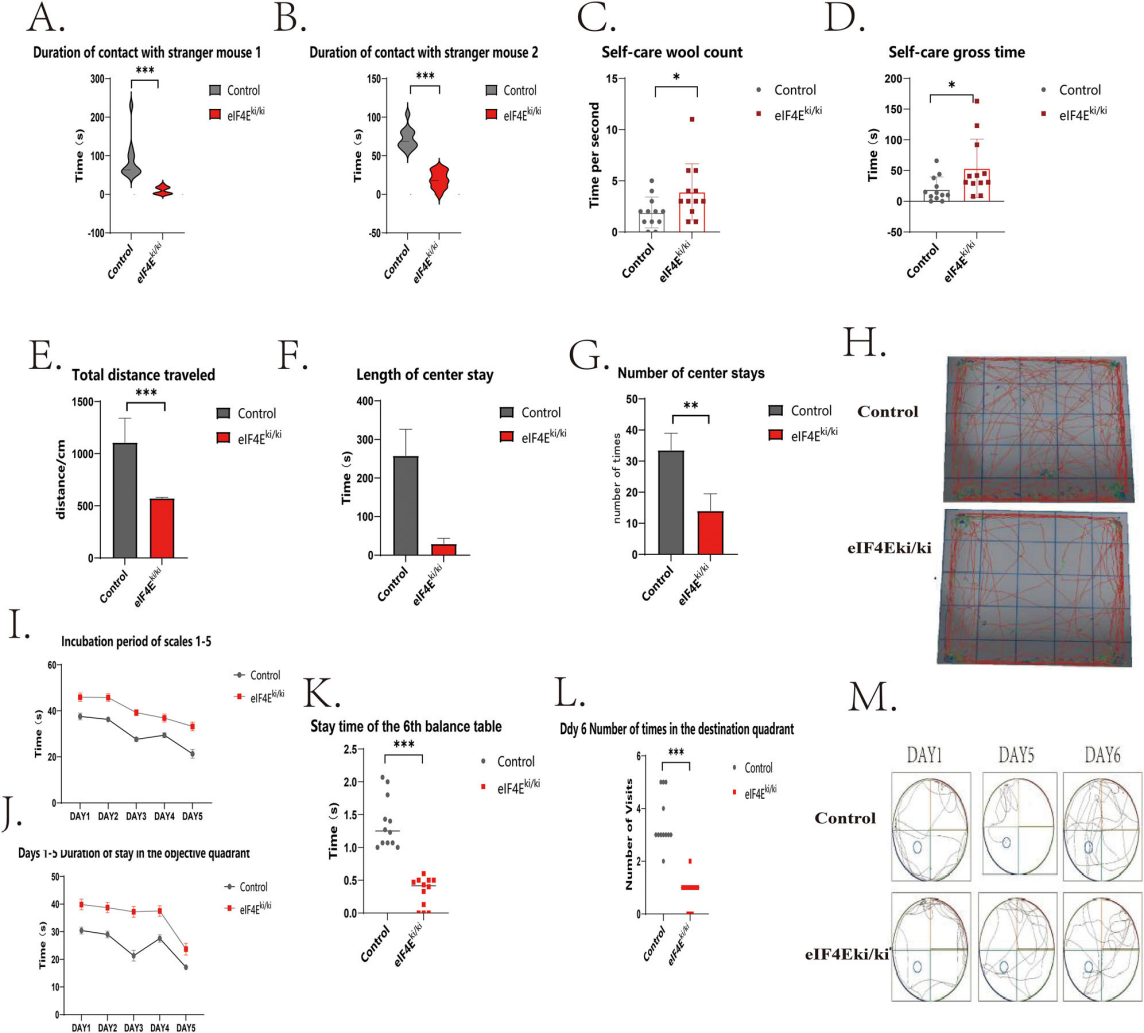
The effect of *eIF4E* overexpression on social cognitive behavior in mice was assessed. ***A***, In the first phase of the three-box social experiment, the duration of contact time between control and eIF4E^ki/ki^ mice and Stranger Mouse 1 was measured. ***B***, In the second phase of the three-box social experiment, the duration of contact between control and eIF4E^ki/ki^ mice and Stranger Mouse 2 was recorded. ***C***, ***D***, The objective of the self-care experiments was to quantify the time spent on self-care activities and the number of self-care procedures performed by control and eIF4E^ki/ki^ mice. ***E–G***, Open-field assays were conducted to assess the total distance traversed, duration of open-field stays, and number of open-field stays by control and eIF4E^ki/ki^ mice. ***H***, The trajectories of mice in open-field experiments are plotted. ***I–M***, The water maze tests included a 5 d localization navigation phase, during which platform latency and destination quadrant residence time were measured in control and eIF4Ek^ki/ki^ mice. On the sixth day of the experiment, the time spent by the subjects on the platform and the number of visits to the destination quadrant were recorded. ***N***, The trajectories of mice in the Morris water maze test are plotted. The Mann–Whitney *U* test was used to statistically analyze data from the three-chamber social interaction test, open-field test, and self-grooming test, along with the duration spent and the frequency of entries into the target quadrant during the water maze test. Escape latency during the learning phase of the water maze was analyzed using two-way ANOVA followed by Tukey's multiple-comparisons test. Data are presented as mean ± standard deviation, and statistical significance is denoted as *p < 0.05, **p < 0.01, and ***p < 0.0001 (control, *n* = 12; eIF4E^ki/ki^, *n* = 12).

Previous studies have shown that eIF4E overexpression regulates learning and memory functions, as well as repetitive stereotypical behaviors, which are considered to be fundamental aspects of social cognition (Amorim et al., 2018; Wiebe et al., 2020; Hooshmandi et al., 2021). Therefore, we initially paired male and female eIf4E-overexpressing mice obtained from the company to generate offspring. We then proceeded with genetic identification and selected homozygous mice. Subsequently, we conducted a further investigation into the behavioral patterns of these mice at the age of 1 month. In the three-box experiment (Week 9), a significant reduction in exposure time to Stranger 1 was observed in the eIF4E^ki/ki^ mice compared with the control group (*p* = 0.0003, *p* < 0.001; Fig. 1*A*). For further details, the results demonstrate a decline in the social interaction function of the eIF4E^ki/ki^ mice. In the social novelty test stage, the eIF4E^ki/ki^ mice had significantly reduced contact time with Stranger 2 in comparison to the control group (*p* = 0.0003, *p* < 0.001; Fig. 1*B*), indicating that the eIF4E^ki/ki^ mice demonstrated a diminished capacity for social novelty. These findings suggest that the overexpression of eIF4E may contribute to social dysfunction in mice. The self-care experiment, conducted over a 10-week period, revealed a notable increase in the number and frequency of self-care activities among the eIF4E^ki/ki^ group mice when compared with the control group (Fig. 1*C,D*). This observation was statistically significant (*p* = 0.046, *p* < 0.05; *p* = 0.04, *p* < 0.05), indicating that the overexpression of eIF4E may contribute to the exacerbation of repetitive and rigid behavior of the mice. In the open-field test conducted during Week 11, the eIF4E^ki/ki^ mice exhibited a notable reduction in total distance traveled in the open field when compared with the control group (Fig. 1*E*; *p* = 0.0003, *p* < 0.001). The eIF4E^ki/ki^ mice were more active in open fields and corners, repeatedly climbed walls, and showed certain anxiety-related behaviors. The exploratory behavior (the frequency of exploration and the duration of stay in the central region) of eIF4E^ki/ki^ mice was significantly lower than that of control mice (*p* = 0.001; *p* = 0.0003, *p* < 0.001; Fig. 1*F–H*). Overexpression of eIF4E resulted in impaired motor function, anxiety-like behavior, and spatial exploration behavior in mice. Finally, the Morris water maze experiment, conducted during Week 12, studied that employed tasks designed to assess the subjects’ abilities in navigation and exploration of novel environments. Each animal was tested four times per day for 5 consecutive days for the localization navigation experiment (Fig. 1*I–N*).

### Overexpression of eIF4E decreased oxytocin expression in mice’s hippocampus

**Figure 2.**
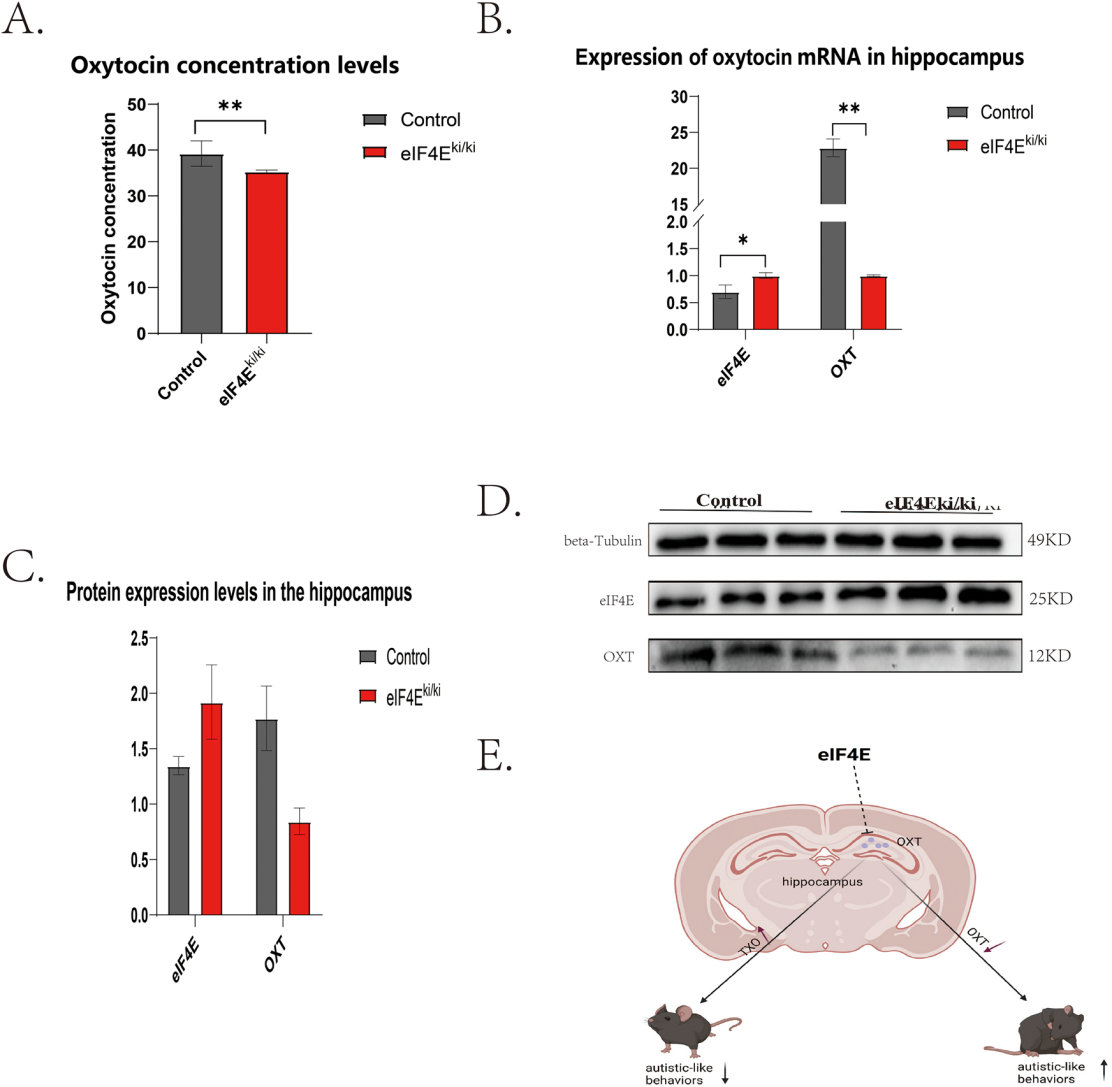
The impact of *eIF4E* overexpression on hippocampal oxytocin levels in mice. ***A***, ELISA experiment reveals the concentrations of hippocampal oxytocin in control and eIF4E^ki/ki^ mice (3 animals in each group). ***B***, qPCR experiment demonstrates the mRNA expression levels of hippocampal oxytocin in control and eIF4E^ki/ki^ mice (3 animals in each group). ***C***, ***D***, Western blot assay displays the protein expression levels of oxytocin in the hippocampus of control and eIF4E^ki/ki^ mice (3 animals in each group). Mann–Whitney *U* test was used for comparison between the two groups. The data are presented as mean ± standard deviation, and statistical significance is denoted as *p < 0.05 and **p < 0.01.

Given the observed alterations in learning and memory function observed in mice, the overexpression of *eIF4E* in the hippocampus may be implicated (Wiebe et al., 2020). It is noteworthy that the hippocampus regulates learning and memory, as well as governing stereotyped memory and social cognitive ability, which are characteristic symptoms of social cognitive behavior associated with autism (Barzegari et al., 2023; Basu et al., 2024). Oxytocin, a pleiotropic neuropeptide present in brain tissue, exerts a pivotal influence on social behavior (P. Fu et al., 2023; Portnova et al., 2023). Its levels are subject to modulation by a range of factors, including time, emotional state, and anxiety-related behaviors (Y-J. Li et al., 2020). In light of these findings, we postulated that the overexpression of *eIF4E* may influence oxytocin expression within the hippocampus. Our ELISA results (Fig. 2*A*) demonstrated that oxytocin levels were significantly lower in the hippocampus of eIF4E^ki/ki^ mice compared with control mice (*p* = 0.006, *p* < 0.01). Furthermore, oxytocin levels were assessed at both mRNA and protein levels using QPCR (Fig. 2*B*) and Western blotting (Fig. 2*C,D*). Consistent with our ELISA findings, there was a significant decrease in oxytocin expression within the hippocampus of eIF4E^ki/ki^ mice (*p* = 0.001, *p* < 0.01; *p* = 0.01, *p* < 0.05). The findings suggest that the overexpression of *eIF4E* results in a reduction in the expression of oxytocin within the mouse hippocampal tissue (Fig. 2*E*).

### The expression of oxytocin in the hippocampus decreases in eIF4E overexpression mice, leading to excessive microglial activation

**Figure 3.**
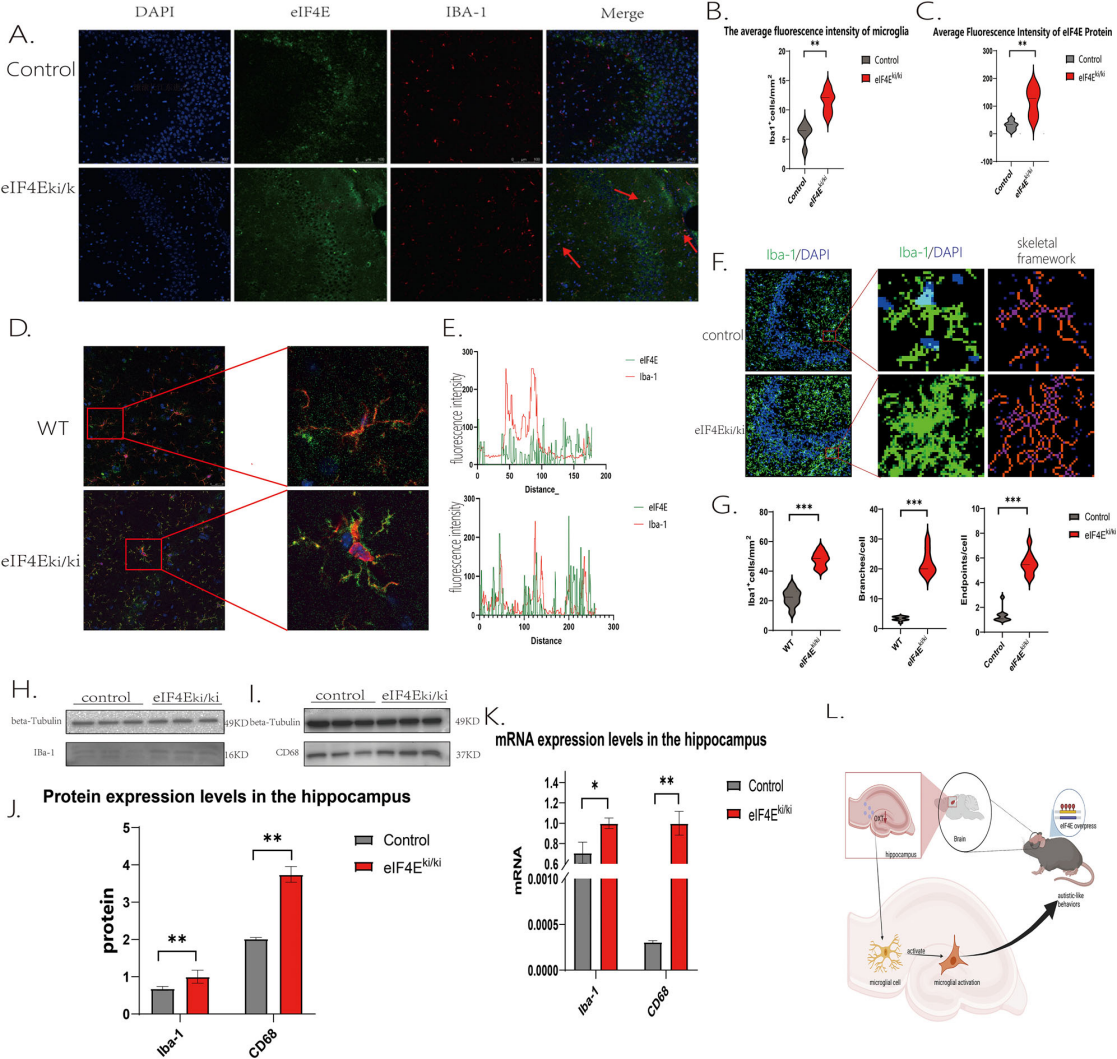
Effect of *eIF4E* overexpression on mouse microglia in immunofluorescence assay. ***A***, Immunofluorescence staining of eIF4E and Iba-1 in hippocampal CA3 region of mice in each group; the arrow indicates the activation state of microglia. ***B***, Comparison of the fluorescence intensity of eIF4E protein expression in the hippocampal CA3 region of mice in each group. ***C***, Comparison of the fluorescence intensity of the expression of the microglial activation marker protein Iba-1 in the hippocampal CA3 region of mice in the two groups. ***D***, Colocalization of eIF4E and microglia in the hippocampal CA3 region of the two groups of mice. ***E***, Analysis of colocalization data of eIF4E and microglia in the hippocampal CA3 region of the two groups of mice. ***F***, ***G***, Western blot assay, expression of Iba-1 protein in the hippocampus of control and eIF4E^ki/ki^ mice. ***H***, qPCR assay. Oxytocin mRNA expression levels in the hippocampus of control and eIF4E^ki/ki^ mice. ***I***, eIF4E overexpression causes autism-like social cognitive impairment. Mann–Whitney *U* test was used for comparison between the two groups. The data are presented as mean ± standard deviation, and statistical significance is denoted as *p < 0.05 and **p < 0.01. In the study, *n* refers to the number of animals, with five acquisitions from each (hippocampus) slice, with a maximum of three slices obtained from each experimental animal used for each protocol (3 animals in each group).

The anti-inflammatory and antioxidant properties of oxytocin in vivo, its ability to regulate the immune and anti-inflammatory responses of microglia in the brain and ameliorate autism-like abnormal behaviors (Malishkevich et al., 2015; Maejima et al., 2022), prompted us to investigate whether the reduced expression in the hippocampus is associated with aberrant microglial function. Immunofluorescence analysis revealed colocalization staining results for Iba-1 and eIF4E proteins (Fig. 3*A*). In comparison to the control group, eIF4E^ki/ki^ mice exhibited a lack of detectable eIF4E protein in the CA3 region of the hippocampus (Fig. 3*B*; *p* = 0.007, *p* < 0.01). Additionally, the fluorescence intensity of Iba-1 was significantly elevated (Fig. 3*C*; *p* = 0.0017, *p* < 0.01), and the distribution density of Iba-1 protein was notably higher in eIF4E^ki/ki^ mice (Fig. 3*G*). Colocalization analysis further revealed that the fluorescence intensity distribution between the two proteins was more consistent in eIF4E^ki/ki^ mice compared with control mice (Fig. 3*D,E*). These findings suggest a significant upregulation of both proteins within the hippocampus, with clear colocalization observed in this region. Further morphological analysis of microglial cells indicated that relative to the control group, eIF4E^ki/ki^ mice exhibited significantly enlarged cell bodies (Fig. 3*F*). Moreover, the number of branches proximal to the microglial cell bodies was significantly increased in the eIF4E^ki/ki^ group (*p* = 0.0041, *p* < 0.01), as was the number of terminal branches (*p* = 0.0041, *p* < 0.01; Fig. 3*F,G*). These results suggest that the microglial cells transitioned from a simple “ameboid” state to a highly branched “ramified ameboid” state, reflecting a shift from a quiescent to a highly activated morphology known as “hyper-ramification” (Walker et al., 2014; Smith et al., 2019). Western blot and qPCR analyses confirmed an increase in the protein levels of Iba-1 and CD68 markers (*p* = 0.004, *p* < 0.01; *p* = 0.04, *p* < 0.05), as well as elevated mRNA expression levels (*p* = 0.003, *p* < 0.01; *p* = 0.02, *p* < 0.05). These findings indicate that the overexpression of eIF4E stimulates microglial activation within the hippocampal regions of mice.

## Discussion

The initiation factor of protein translation,* eIF4E*, has emerged as a prominent area of focus in ASD research due to its pivotal role in regulating mRNA translation (Gkogkas et al., 2013; D. Liu et al., 2022). It has been demonstrated that mutations in *eIF4E* are associated with aberrant protein synthesis, which may contribute significantly to the manifestation of ASD-like behaviors (Camargo et al., 2022). This study used mice overexpressing *eIF4E* as a model system to conduct behavioral tests. The findings revealed that the *eIF4E* overexpression mice exhibited a reduced preference for the central region in comparison with the control group during the open-field test.

Furthermore, the mice exhibited augmented self-care behavior, both in terms of quantity and temporality, when compared with their control counterparts. These observations align with previous studies by Ke et al. (2023), suggesting that elevated levels of *eIF4E* may be implicated in anxiety-related cognitive impairments (Yu et al., 2023). The Morris water maze test results demonstrated that mice overexpressing *eIF4E* exhibited prolonged latency during learning and reduced residence time in the target quadrant, thereby confirming impaired learning and memory abilities associated with *eIF4E* overexpression. Previous studies have indicated that elevated levels of *eIF4E* can impact the cognitive functions of mice, potentially due to aberrant hippocampal neuronal and synaptic activity (Santini et al., 2013; Ke et al., 2023).

Additionally, the social capabilities of *eIF4E*-overexpressing mice were evaluated using a three-box social test. In both the social ability and social novelty stages, these mice spent less time engaging with unfamiliar counterparts than control mice. This indicates that compromised sociability results from eIF4E overexpression. This finding aligns with the observations of Santini, who observed that genetically increasing *eIF4E* levels in mice lead to excessive cap-dependent translation, repetitive stereotypic behavior patterns, and deficits in social interactions (Allely et al., 2024). In conclusion, our findings suggest that eIF4E overexpression induces the emergence of ASD-like behaviors, particularly those resembling social cognitive impairments.

A considerable body of previous research has consistently demonstrated that social interaction ability and social cognitive behavior are crucial factors in establishing and maintaining social relationships (Allely et al., 2024; Lievore et al., 2024). Oxytocin, a neuropeptide, is significant in regulating social behavior, particularly social cognitive behavior. The hippocampus is a vital center for the regulation of social cognitive function and is critically involved in brain learning and memory processes (Tudor et al., 2016; Aboubakr et al., 2023; Y. Zhang et al., 2024). This study investigates the potential association between abnormal oxytocin levels in the hippocampus and the social cognition impairments observed in individuals with ASD. Previous studies have indicated that overexpression of *eIF4E* can lead to hormone protein synthesis, which may affect hormone expression levels (Song et al., 2023; Y. Zhang et al., 2024). In this study, an ELISA test was conducted to measure oxytocin expression levels in the hippocampus of mice with *eIF4E* overexpression. The results revealed a decrease in oxytocin expression levels in the test subjects when compared with control mice. Western blotting and qPCR analysis further confirmed reduced oxytocin mRNA and protein levels within the hippocampus. The findings of this study suggest that *eIF4E* overexpression may contribute to decreased oxytocin expression within the hippocampus, which could contribute to impaired social cognition observed in these mice.

It is noteworthy that the level of serum oxytocin was found to be lower than that observed in the hippocampus. This finding is consistent with the results of previous studies (Ye et al., 2022; Yonekura et al., 2023). The Western blot and qPCR analyses demonstrated a significant decrease in the expression of oxytocin at both mRNA and protein levels within the hippocampus. These findings suggest that overexpression of *eIF4E* may reduce oxytocin levels, particularly within the hippocampus, which may contribute to social cognitive impairment observed in mice with elevated eIF4e expression.

A growing body of research has demonstrated that autism is associated with neuroinflammation and neurotransmitter abnormalities (Chen et al., 2024). Microglia, the brain's resident immune cells responsible for initiating neuroinflammatory responses, constitute approximately 10% of the glial cell population in the cerebral cortex (Cohen et al., 2024). Both clinical and animal studies have reported that microglial activation-induced neuroinflammation can result in ASD-like behaviors (M. Yang et al., 2023; Borreca et al., 2024). Immunofluorescence-science assay revealed a significant increase in fluorescence intensity and distribution density of Iba-1, an activation marker for microglia, within the hippocampal CA3 region of *eIF4E*-overexpressing mice compared with the control group. The observation that the cell bodies of microglia in the hippocampus enlarged, the number of branches increased, and they presented a highly branched “ameba”-like shape indicates the excessive activation of astrocytes. Localization analysis revealed that the colocalization of the *eIF4E* protein with Iba-1 was significantly higher in *eIf4E*-overexpressing mice compared with control mice, indicating a significant increase in *eIF4E* protein expression on microglia in the hippocampus. This finding suggests that overexpression of *eIF4E* enhances both the density and size of microglia and their phagocytic ability. These findings are consistent with those reported by Xu et al. (2020). Oxytocin, a pleiotropic neuropeptide, has been shown to possess anti-inflammatory properties and protect the neural microenvironment (Knoop et al., 2022). Previous studies have demonstrated that oxytocin can inhibit microglial activation by suppressing the pathway, attenuating neu-neuroinflammation, and improving social cognition (B’Chir et al., 2013). It has been reported that decreased oxytocin expression due to mutations in autism susceptibility genes Cntnap2 and Shank3b has been observed to microglial activation (M. Yang et al., 2023; Yuan et al., 2016) Overexpression of *eIF4E* can result in abnormal protein synthesis and promote neu-neuroinflammation through microglial activation. Conversely, downregulation of *eIF4E* expression can reduce microglial activation and inhibit neuroinflammation (D. Liu et al., 2022). In this study, Western blotting and qPCR analysis demonstrated an upregulation of Iba-1 and CD68 expression (markers for activated microglia) in the hippocampus of mice overexpressing *eIF4E*. This suggests that overexpression of *eIF4E* may induce neuroinflammation, which is characterized by excessive activation of microglia in the hippocampus. Considering our finding that overexpression of *eIF4E* led to decreased oxytocin expression levels, we hypothesize that the social cognitive impairment observed in these mice may be associated with neuroinflammation resulting from reduced oxytocin expression.

The present study demonstrated that the overexpression of *eIF4E* in mice induces social cognitive impairment, which may be attributed to the excessive activation of microglia resulting from reduced oxytocin levels. However, it should be noted that no intervention was conducted on the *eIF4E*-overexpressing mice during the course of the experiment. Although microglial activation in the hippocampus of *eIF4E*-overexpressing mice is hyperactive, the present study has not confirmed whether increasing oxytocin expression to regulate microglial hyperactivation can ameliorate autistic behaviors in these mice. Therefore, further experiments are warranted to investigate whether the regulatory effects of oxytocin on microglial function can improve autism-like social cognitive impairment.

## Conclusion

The findings reveal that oxytocin plays a pivotal role in modulating the functional state of microglia during the development of autism, particularly in individuals associated with *eIF4E* mutation. These findings will provide unique insights into the molecular mechanisms underlying autistic behavior and facilitate the identification of more precise therapeutic targets for autism.

## Synthesis

Reviewing Editor: Eran Perlson, Tel Aviv University Decisions are customarily a result of the Reviewing Editor and the peer reviewers coming together and discussing their recommendations until a consensus is reached. When revisions are invited, a fact-based synthesis statement explaining their decision and outlining what is needed to prepare a revision will be listed below. The following reviewer(s) agreed to reveal their identity: Vivig Shantha Kumar.

This manuscript offers a novel and intriguing exploration of the mechanisms linking eIF4E overexpression to autism spectrum disorder (ASD)-like behaviors, particularly through microglial activation and reduced oxytocin levels. While EIF4E overexpression was reported to induce autism spectrum disorders (ASD) like behavior deficits, depression-like behavior and cognitive impairment (Christos G. Gkogkas et al., 2012; Thu N. Huynh et al., 2015; Argel Aguilar-Valles et al., 2018)., the mechanisms are not fully explored. In the current work, authors first repeated the autism-like social cognitive deficits in eIF4E overexpression mice with different behavior tests. Further, they found that eIF4E overexpression induced oxytocin expression down regulation in hippocampus due to microglia hyperactivation.

While the study has potential, several important clarifications and experimental steps are needed before it can be considered for publication:

1, eIF4E overexpression reduced oxytocin expression. Does intracerebral ventricular injection of oxytocin rescue the behavioral deficits?

2, Oxytocin is released by PVN OXT neurons. eIF4E is highly expressed in neurons. Does eIF4E overexpression change PVN OXT neurons activity?

3, Authors propose that oxytocin downregulation in hippocampus induce microglia activation and further results in autistic like behavior changes (Fig.3I). Iba1 mRNA level or protein level changes is not good enough to claim microglia activation. Authors need to measure microglia density and morphology changes, and also include other markers, such as CD68 and MHCII.

If microglia activation mediates ASD like behavior, how does microglia depletion change these behaviors in eIF4E overexpression mice?

4, Fig1e shows that eIF4E overexpression mice traveled far less than WT control mice. Does eIF4E overexpression mice have motor deficits? If so, does motor deficits contribute to reduced contact time to stranger mice in Fig1a-b?

5, In Fig1m, eIF4Eki/ki mice show around one third of time in the destination quadrant compared to WT mice. However, the example traces in Fig1n does not support that. What is the Y axis lab in Fig1m?

6, Statistical methods should be include in figure legends for each panel.

Minor points:

1, Line 242 to 243, "...wild group..." mean wild type littermate control group? Change "wild group" expression in the context.

2, Line 632, change "stranger 1 mouse 1".

## Author Response

Dear Editors and Reviewers:

Thank you for your letter and the reviewers' comments on our manuscript entitled "The overexpression of eIF4E decreases oxytocin levels and induces social-cognitive behavioral disorders in mice". These comments are valuable and helpful for us to revise and improve the paper, as well as significance guidance for our research. We have carefully studied these comments and made revisions in the hope that they will be recognized. The revised parts are marked in red in the paper. The main revisions in the paper and the responses to the reviewers' comments are listed below:

Reviewer :

1, eIF4E overexpression reduced oxytocin expression. Does intracerebral ventricular injection of oxytocin rescue the behavioral deficits? Author response:Thank you for your valuable feedback. After reviewing the relevant literature(Yin, et al. 2024, Yan, et al. 2014, Kovács and Telegdy 1983)，we found that intraventricular injection of oxytocin has demonstrated positive behavioral effects in mouse models of depression and brain hemorrhage . However, there is currently a lack of research on intraventricular injection in mouse models of autism. Your suggestion is innovative and provides a promising direction for future research. Nevertheless, the clinical implementation of intraventricular drug delivery poses significant challenges. Therefore, under ethical premises, we will further investigate the potential therapeutic effects of exogenous oxytocin in animal models (such as eIF4E overexpressing mice). This will help elucidate the impact of oxytocin on behavioral defects caused by eIF4E overexpression and contribute to a better understanding of its potential application in clinical intervention. We greatly appreciate your feedback and will actively consider delving into this topic in future research.

2, Oxytocin is released by PVN OXT neurons. eIF4E is highly expressed in neurons. Does eIF4E overexpression change PVN OXT neurons activity? Author response:Thank you for your valuable feedback. According to previous literature(Yang, et al. 2024, Gong, et al. 2024), the abnormal behavior exhibited by eIF4E overexpressing mice may not be directly related to the release ability of PVN oxytocin neurons, but rather to the damage of neurons in various brain areas affecting their ability to receive oxytocin. In our preliminary experiment, we performed HE staining on the whole brain of eIF4E mice to explore the extent of neuronal damage in various brain regions. As shown in the figure below, we only observed damage to neurons in the hippocampal CA3 region (nuclear condensation, deep blue-black staining, and shrinkage of cell bodies), and did not find any other damaged areas. We believe that this neuronal damage is a significant factor leading to social cognitive impairments in eIF4E mice and will be the focus of our next research direction.

3, Authors propose that oxytocin downregulation in hippocampus induce microglia activation and further results in autistic like behavior changes (Fig.3I). Iba1 mRNA level or protein level changes is not good enough to claim microglia activation. Authors need to measure microglia density and morphology changes, and also include other markers, such as CD68 and MHCII.

If microglia activation mediates ASD like behavior, how does microglia depletion change these behaviors in eIF4E overexpression mice? Author response:In response to your suggestion, we have added a detailed analysis of the density and morphology of microglia to the revision. Specifically, we quantified the density and morphology of microglia by 3D imaging and skeleton analysis. In addition, we supplemented the measurements of protein and mRNA levels of CD68 to assess the activation status of microglia more fully. We believe these supplementary experiments and data can provide more robust support for the study's conclusions and meet your requirements.

Thank you for raising the critical question of the potential role of microglia in the behavior of eIF4E-overexpressing mice. We fully recognize the important role of microglia in maintaining normal function and neural homeostasis in the central nervous system, and their activation status is closely related to pathological conditions.

1.The physiological function of microglia is critical: Microglia not only play a key role in neural development but also play an essential role in synaptic plasticity, neuroprotection, and immune surveillance. Loss of microglia may lead to dysregulated neural networks, abnormal synaptic development, and even global behavioral patterns. In the study, we wanted to preserve the normal physiological function of microglia to more closely mimic the actual state of the nervous system and avoid the complex confounding effects that the absence of these cells may introduce.

2. Role of microglial activation in disease models: We focused on regulating eIF4E overexpression on microglial activation and its role in ASD-like behaviors. Activated microglia may have neuronal and behavioral effects by releasing proinflammatory factors and modulating synaptic pruning. Therefore, we believe that maintaining the activated state of microglia better mimics the neuropathological features in ASD and helps reveal the role of microglia in neuroinflammation and behavioral changes.

3. Avoiding additional effects of microglial depletion: The potential for new behavioral abnormalities and neurological deficits to arise from microglial depletion will complicate the findings and make it difficult to clearly distinguish the behavioral deficits caused by eIF4E overexpression from the effects of microglial depletion.

Therefore, we did not perform microglial depletion experiments to maintain the clarity of the experimental design and the clarity of the conclusions.

We hope this explanation sheds light on our experimental design choices and look forward to further discussions with you.

4, Fig1e shows that eIF4E overexpression mice traveled far less than WT control mice. Does eIF4E overexpression mice have motor deficits? If so, does motor deficits contribute to reduced contact time to stranger mice in Fig1a-b? Author response:Thank you for pointing out the potential relevance of reduced travel in eIF4E-overexpressing mice (Figure 1e) to their social behavior tests (Figure 1a-b). Our views on this issue are as follows:

First, the reduced travel in the eIF4E-overexpressing mice in Figure 1e suggests that there is indeed some alteration in motor behavior. We do not think that this is a motor defect (i.e., a decrease in motor coordination or muscle control), because the reduced distance run may reflect other factors, such as a decrease in autism-like active exploration, which we did not find in the eif4E-overexpressing mice in the water-maze test.

We hope that this explanation will clarify the relationship between changes in exercise behavior, and social behavior outcomes and appreciate your valuable comments.

5, In Fig1m, eIF4Eki/ki mice show around one third of time in the destination quadrant compared to WT mice. However, the example traces in Fig1n does not support that. What is the Y axis lab in Fig1m Author response:We understand your doubts about the consistency between the statistical results in Figure 1M and the trajectory plots in Figure 1N. Here is our explanation:

1. Representatives of the trajectory map: The trajectory map in Figure 1N is based on representative data of individual mice, while Figure 1M reflects the results of data analysis of the whole experimental group. As a qualitative presentation tool, the trajectory map may not adequately represent the average behavior of the entire experimental group. Thus, while the trajectories of individual mice appear to be close, the overall statistical results show significant group differences.

2. Robustness of statistical analysis: The data in Figure 1M are based on the average behavior of multiple mice. After rigorous statistical tests, the results show that eIF4Eki/ki mice spend significantly less time in the target quadrant (about one-third of control mice). We believe these data reliably reflect differences between the two groups of mice. If necessary, we are willing to provide further trajectory maps showing the behavioral characteristics of the entire experimental group to better support the statistical results in Figure 1M. We are more than willing to make changes if additional suggestions are made or further clarifications are needed.

6, Statistical methods should be include in figure legends for each panel.

Author response:Thank you for your valuable comments on the statistical methods in our manuscript. Based on your suggestion, we have modified the legends of each figure and now include detailed descriptions of statistical methods in the legends of all panels. We believe that these changes have improved the clarity and understanding of the results.

Minor points:

1, Line 242 to 243, "...wild group..." mean wild type littermate control group? Change "wild group" expression in the context.

2, Line 632, change "stranger 1 mouse 1".

Author response:1 We have replaced "wild group" with "wild type peer control group" in the manuscript to ensure more accurate expression.

2. In line 632, we have modified "stranger 1 mouse 1" to" stranger 1 mouse 1" to conform to the expression more accurately.

We tried our best to improve the manuscript and made some changes in the manuscript. These changes do not influence the content and framework of the paper. And here we have marked them in red in the revised paper.

We would like to express our sincere gratitude to the editors/reviewers for their dedicated work and hope that corrections will be recognized.

Thanks again for your comments and suggestions.

Gong, Qichao, Tahir Ali, Yue Hu, Ruyan Gao, Shengnan Mou, Yanhua Luo, Canyu Yang, et al. "Ripk1 Inhibition Mitigates Neuroinflammation and Rescues Depressive-Like Behaviors in a Mouse Model of Lps-Induced Depression." Cell Communication and Signaling 22, no. 1 (2024): 427.

Kovács, Gábor L, and Gyula Telegdy. "Effects of Oxytocin, Des-Glycinamide-Oxytocin and Anti-Oxytocin Serum on the Α-Mpt-Induced Disappearance of Catecholamines in the Rat Brain." Brain research 268, no. 2 (1983): 307-14.

Yan, Yuan, Yong-Liang Wang, Zhou Su, Yi Zhang, Shuang-Xi Guo, Ai-Juan Liu, Chang-Hong Wang, Fang-Jie Sun, and Jun Yang. "Effect of Oxytocin on the Behavioral Activity in the Behavioral Despair Depression Rat Model." Neuropeptides 48, no. 2 (2014): 83-89.

Yang, Canyu, Tahir Ali, Axiang Li, Ruyan Gao, Xiaoming Yu, Shupeng Li, and Tao Li. "Ketamine Reverses Chronic Corticosterone-Induced Behavioral Deficits and Hippocampal Synaptic Dysfunction by Regulating Eif4e/Bdnf Signaling." Neuropharmacology (2024): 110156.

Yin, Hailian, Meiyun Jiang, Tao Han, and Xiaolei Xu. "Intranasal Oxytocin as a Treatment for Anxiety and Autism: From Subclinical to Clinical Applications." Peptides (2024): 171211
